# Improving Healthy Behavior and Adopting Healthy Habits by Bodywise Program for Older Adult DC Residents

**DOI:** 10.1093/geroni/igaa057.1297

**Published:** 2020-12-16

**Authors:** Lillie Monroe-Lord, Azam Ardekani, James Lee, Elgloria Harrison

**Affiliations:** 1 University of the District of Columbia, Washington, District of Columbia, United States; 2 Howard University, Beltsville, Maryland, United States

## Abstract

An increase in physical activity (PA) level is associated with reduction in chronic diseases. However, the number of older adults who meet the PA guideline is low and decreases with age. Bodywise is a free program that aims to promote healthy behavior and to encourage older adults who live in Washington, DC to adopt a healthy habit by increasing their PA. The Bodywise program offers water aerobics, yoga, low-impact aerobics, and chair exercise classes, conducted by trained instructors at the University of the District of Columbia. This study reports an evaluation of the implementation of this program based on the surveys completed by participants after finishing the classes. Participants were DC residents aged 60 or older. According to the surveys, 68% of participants have adopted one or more healthy habits since joining the Bodywise program. In addition, more than 62% of participants have an increase in awareness of healthy behavior through this program. Overall satisfaction with the Bodywise program is 93%, satisfaction with the variety of classes offered is 72% and satisfaction with offered skill levels is 75%. Furthermore, 81% of participants reported overall satisfaction with their instructor, specifically 100% satisfaction related to their knowledge and skill level, and appropriateness of selected music, moves, and exercises. In conclusion, the Bodywise program leads to an overall change in both awareness of healthy behavior and development of healthy habits in participants. Further research can investigate the correlation of health awareness and healthy habits with overall satisfaction with the program and the instructor.

